# Supplementary material for: The indirect effect of mRNA-based COVID-19 vaccination on healthcare workers’ unvaccinated household members
Source: Nat Commun. 2022 Mar 4;13:1162. doi: 10.1038/s41467-022-28825-4 (PMC8897446; doi:10.1038/s41467-022-28825-4)
Supplement: Supplementary file 1 — Supplementary Information [file 41467_2022_28825_MOESM1_ESM.pdf]

Supplementary Information for

The indirect effect of mRNA-based COVID-19 vaccination on healthcare workers' unvaccinated household members

Jussipekka Salo[1,2,3,4], Milla Hägg[1,2,3,4], Mika Kortelainen[5,6, 2,4] Tuija Leino[7],

Tanja Saxell[2,4], Markku Siikanen[2,4], Lauri Sääksvuori[8,9,10]*

Contents

A.) Supplementary Tables……………………………………………….......2

B.) Supplementary Figures……..…………………………………………..11

C.) Supplementary Methods…………………………………………….…...14

---

[1]Department of Economics, University of Helsinki, Arkadiankatu 7, P.O. Box 17, 00014 University of Helsinki, Helsinki, Finland. [2]VATT Institute for Economic Research, Arkadiankatu 7, P.O. Box 1279, 00101 Helsinki, Helsinki, Finland. [3]Aalto University, Department of Economics, Aalto University School of Business, P.O. Box 21210, 00076 Aalto, Espoo, Finland. [4]Helsinki GSE, Arkadiankatu 7, 00100 Helsinki, Helsinki, Finland, [5]Department of Economics, Turku School of Economics, Rehtorinpellonkatu 3, 20500 Turku, Turku, Finland, [6]InFLAMES Research Flagship Center, University of Turku, Turku, Finland. [7]Finnish Institute for Health and Welfare, Infectious Disease Control and Vaccinations Unit, P.O. Box 30, 00271 Helsinki, Helsinki, Finland. [8]Tampere University, Health Science Unit, Arvo Ylpön katu 34, 33520 Tampere, Tampere, Finland. [9]University of Turku, INVEST Research Flagship Center, Assistentinkatu 7, 20014 University of Turku, Turku, Finland. [10]Finnish Institute for Health and Welfare, Centre for Health and Social Economic, P.O. Box 30, 00271 Helsinki, Helsinki, Finland. Correspondence should be addressed to Lauri Sääksvuori (lauri.saaksvuori@thl.fi).

A Supplementary Tables

Supplementary Table 1 Healthcare worker occupation codes.

Occupation code	Included occupations	Excluded occupations (codes)
221	Physicians	
222	Senior nursing officials, Ward sisters	
322	Nurses, Midwifes	
226	Dentists, Audiologists and Speech therapists	Pharmacists (22620), Environmental and occupational health and hygiene professionals (22630), Dieticians and nutritionists (22650), Health professionals not elsewhere classified (22690)
532	Practical nurses	Pharmaceutical assistants (53293), Equipment maintenance assistants (53292), Massage therapists and practical rehabilitation nurses (53294)

Notes: This table shows the occupations we use to define healthcare workers in our sample. Some occupations are excluded, because a significant share of individuals in these occupations may work outside the healthcare sector. Occupation codes are based on Statistics Finland's classification of occupations (TK10).

Our healthcare worker definition was based on the occupation classification of Statistics Finland (TK10). Supplementary Table 1 shows the occupations which we use to define healthcare workers in our sample. We used 3-digit occupation codes to identify healthcare professions. Some 5-digit occupation codes within these 3-digit codes were excluded, because a significant share of individuals within these occupations may work outside the healthcare sector.

Supplementary Table 2 Descriptive statistics for healthcare workers (HCW) and their partners.

a	Vaccinated HCWs (N = 112 496)	Unvaccinated HCWs (N = 152 830)
Age – Mean (SD)	48.2 (13.81)	41.5 (13.49)
Women – N (%)	97 125 (86.34)	132 695 (86.83)
Household size – N (%)		
1	21 567 (19.17)	29 538 (19.33)
2	44 565 (39.61)	51 039 (33.40)
3	18 554 (16.49)	28 642 (18.74)
4	17 895 (15.91)	26 043 (17.04)
5+	9 915 (8.81)	17 568 (11.50)
Ethnicity – N (%)		
Finnish	107 258 (95.34)	141 239 (92.42)
Foreign	5 238 (4.66)	11 591 (7.58)
Geographic region – N (%)		
Urban	76 875 (68.34)	110 380 (72.22)
Semi-urban	18 481 (16.43)	24 551 (16.06)
Rural	17 140 (15.24)	17 899 (11.71)

b	Partners of vaccinated HCWs (N = 49 219)	Partners of unvaccinated HCWs (N = 79 733)
Age – Mean (SD)	47.1 (11.96)	43.7 (12.23)
Women – N (%)	5 430 (11.03)	8 628 (10.82)
Household size – N (%)		
2	19 375 (39.36)	30 432 (38.17)
3	9 897 (20.11)	17 156 (21.52)
4	12 699 (25.80)	19 256 (24.15)
5+	7 248 (14.73)	12 889 (16.17)
Ethnicity – N (%)		
Finnish	46 928 (95.35)	73 911 (92.70)
Foreign	2 291 (4.65)	5 822 (7.30)
Geographic region – N (%)		
Urban	32 317 (65.66)	54 911 (68.87)
Semi-urban	8 800 (17.88)	14 319 (17.96)
Rural	8 102 (16.46)	10 503 (13.17)

Notes: This table presents descriptive statistics for the vaccinated and unvaccinated healthcare workers and their partners. **a** shows descriptive statistics for healthcare workers. **b** shows descriptive statistics for partners.

Supplementary Table 3 Descriptive statistics for children by age group.

a	Children of vaccinated HCWs (N = 64 419)	Children of unvaccinated HCWs (N = 104 729)
Age – Mean (SD)	11.04 (4.41)	10.40 (4.47)
Girls – N (%)	31 467 (48.85)	50 415 (48.14)
Household size – N (%)		
2	2 451 (3.80)	4 925 (4.70)
3	10 460 (16.24)	18 980 (18.12)
4	26 072 (40.47)	37 507 (35.81)
5+	25 436 (39.49)	43 317 (41.36)
Ethnicity – N (%)		
Finnish	62 139 (96.46)	98 142 (93.71)
Foreign	2 280 (3.54)	6 597 (6.29)
Geographic region – N (%)		
Urban	42 071 (65.31)	70 015 (66.85)
Semi-urban	11 996 (18.62)	20 315 (19.40)
Rural	10 352 (16.07)	14 399 (13.75)
b	Children of vaccinated HCWs (N = 37 791)	Children of unvaccinated HCWs (N = 67 395)
Age – Mean (SD)	7.93 (2.87)	7.63 (2.83)
Girls – N (%)	18 370 (48.61)	32 370 (48.03)
Household size – N (%)		
2	1 233 (3.26)	2 926 (4.34)
3	5 772 (15.27)	12 401 (18.40)
4	16 138 (42.70)	25 268 (37.49)
5+	14 648 (38.76)	26 800 (39.77)
Ethnicity – N (%)		
Finnish	36 314 (96.09)	62 970 (93.43)
Foreign	1 477 (3.91)	4 425 (6.57)
Geographic region – N (%)		
Urban	25 188 (66.65)	45 405 (67.37)
Semi-urban	6 833 (18.08)	12 870 (19.10)
Rural	5 770 (15.27)	9 120 (13.53)
c	Children of vaccinated persons (N = 26 628)	Children of unvaccinated persons (N = 37 334)
Age – Mean (SD)	15.46 (1.69)	15.39 (1.70)
Girls – N (%)	13 097 (49.19)	18 045 (48.33)
Household size – N (%)		
2	1 218 (4.57)	1 999 (5.35)
3	4 688 (17.61)	6 579 (17.62)
4	9 934 (37.31)	12 239 (32.78)
5+	10 788 (40.51)	16 517 (44.24)
Ethnicity – N (%)		
Finnish	25 825 (96.98)	35 172 (94.21)
Foreign	803 (3.02)	2 162 (5.79)
Geographic region – N (%)		
Urban	16 883 (63.40)	24 610 (65.92)
Semi-urban	5 163 (19.39)	7 445 (19.94)
Rural	4 582 (17.21)	5 279 (14.14)

Notes: This table presents individual level descriptive statistics for healthcare workers' (under 18) children. **a** shows descriptive statistics for children and adolescents aged from 3 to 18 years. **b** shows descriptive statistics for children and adolescents aged from 3 to 12 years. **c** shows descriptive statistics for adolescents aged from 3 to 18 years.

Supplementary Tables 2 and 3 document descriptive statistics for the estimation samples and show that 42% of the healthcare workers were vaccinated and 58% were unvaccinated during the observation period. All variables were well balanced between the treatment and control groups. The mean age of the vaccinated healthcare workers was 48 years (SD 14) and for the unvaccinated controls it was 42 years (SD 13). The mean age was 47 years (SD 12) for the treated partners of vaccinated healthcare workers, and for the controls (the partners of unvaccinated healthcare workers) it was 44 years (SD 12). The mean age for the children of vaccinated healthcare workers was 11 years (SD 4) and for the children of unvaccinated healthcare workers the mean age was 10 years (SD 5). As expected, healthcare workers were typically female (Supplementary Table 2, a) and their partners male (Supplementary Table 2, b) and the sex of the children is evenly balanced (Supplementary Table 3, a). Moreover, over 90% of healthcare workers are Finnish, rather than foreign, and approximately 70% live in urban regions. The sample sizes for the partners and children are smaller than the sample for the healthcare workers. The reasons for this are that some (19%) healthcare workers do not have any family members (household size equals to 1) or the partner or the children are vaccinated (and hence, they are not in our estimation sample of unvaccinated family members).

Supplementary Table 4 Number of healthcare workers and their vaccination status by occupation code.

Occupation	Practical nurses (N=176 289)	Nurses and midwifes (N=65 389)	Physicians (N =15 940)	Dentists, audiologists and speech therapists (N=4065)	Senior nursing officials and ward sisters (N=3643)
Share in sample (%)	66.44	24.64	6.01	1.53	1.37
Age – mean (SD)	44.26 (15.00)	44.12 (11.72)	44.40 (12.20)	44.72 (12.46)	51.92 (9.75)
Women (%)	153070 (86.83)	60 555 (92.61)	9 721 (60.98)	3 168 (77.93)	3 306 (90.75)
Vaccinated (%)	65 288 (37.03)	33 518 (51.26)	10 286 (64.53)	1 415 (34.81)	1 989 (54.60)
Fully vaccinated (%)	32 359 (18.36)	22 051 (33.72)	7 922 (49.70)	456 (11.22)	1 198 (32.89)

Notes: This table presents number of healthcare workers and their age, sex and vaccination status by occupation code. Fully vaccinated means double vaccinated.

Supplementary Table 4 further shows that 25% of healthcare workers were nurses and midwives (occupation code 322), and 6% were physicians (occupation code 221). The analysis sample for healthcare workers mainly consists of practical nurses (occupation code 532) and nurses and midwifes (occupation code 322). There are some differences in vaccination rates between different occupations. The shares of partially and fully (double) vaccinated persons were the highest for physicians (65% and 50%) and the lowest for dentists, audiologists and speech therapists (35% and 11% - occupation code 226).

Supplementary Table 5 Number of infections for healthcare workers (HCW) and their partners.

a	Vaccinated HCWs (N = 321)	Unvaccinated HCWs (N = 1 150)
Women – N (%)	276 (85.98)	985 (85.65)
Men – N (%)	45 (14.02)	165 (14.35)
Household size – N (%)		
1	61 (19.00)	209 (18.17)
2	104 (32.40)	322 (28.00)
3	50 (15.58)	206 (18.91)
4	59 (18.38)	244 (21.22)
5+	47 (14.64)	169 (14.70)
Ethnicity – N (%)		
Finnish	274 (85.36)	848 (73.74)
Foreign	47 (14.64)	302 (26.26)
Geographic region – N (%)		
Urban	269 (83.80)	986 (85.74)
Semi-urban	25 (7.79)	95 (8.26)
Rural	27 (8.41)	69 (6.00)

b	Parners of vaccinated HCWs (N = 262)	Partners of unvaccinated HCWs (N = 520)
Women – N (%)	28 (10.69)	68 (13.08)
Men – N (%)	234 (89.31)	452 (86.92)
Household size – N (%)		
2	108 (41.22)	155 (29.80)
3	39 (14.89)	109 (20.96)
4	65 (24.81)	145 (27.88)
5+	50 (19.08)	111 (21.35)
Ethnicity – N (%)		
Finnish	224 (85.50)	378 (72.69)
Foreign	38 (14.50)	142 (27.31)
Geographic region – N (%)		
Urban	192 (73.28)	428 (82.31)
Semi-urban	37 (14.12)	59 (11.35)
Rural	33 (12.60)	33 (6.35)

Notes: This table presents number of infections for the vaccinated and unvaccinated healthcare workers and their partners. **a** shows number of infections for healthcare workers **b** shows number of infections for partners.

Supplementary Table 6 Number of infections for children by age group.

a	Children of vaccinated HCWs (N = 315)	Children of unvaccinated HCWs (N =505)
Girls – N (%)	167 (53.02)	248 (49.11)
Boys– N (%)	148 (46.98)	257 (50.89)
Household size – N (%)		
2-3	64 (20.31)	120 (23.76)
4	111 (35.24)	186 (36.83)
5+	140 (44.44)	199 (39.41)
Ethnicity – N (%)		
Finnish	274 (86.98)	361 (71.49)
Foreign	41 (13.02)	144 (28.51)
Geographic region – N (%)		
Urban	246 (78.10)	436 (86.34)
Semi-urban	33 (10.48)	45 (9.91)
Rural	36 (11.43)	24 (4.75)
**b**	**Children of vaccinated HCWs (N =173)**	**Children of unvaccinated HCWs (N = 300)**
Girls – N (%)	84 (48.55)	141 (47.00)
Boys– N (%)	89 (51.45)	159 (53.00)
Household size – N (%)		
2-3	31(27.92)	81 (37.00)
4	66 (38.15)	114 (38.00)
5+	76 (43.93)	105 (35.00)
Ethnicity – N (%)		
Finnish	149 (86.13)	205 (68.33)
Foreign	24 (13.87)	95 (31.67)
Geographic region – N (%)		
Urban	131 (75.72)	259 (86.33)
Semi-urban	18 (10.40)	30 (10.00)
Rural	24 (13.87)	11 (3.67)
**c**	**Children of vaccinated persons (N = 142)**	**Children of unvaccinated persons (N = 205)**
Girls – N (%)	83 (58.45)	107 (52.20)
Boys– N (%)	59 (41.55)	98 (47.80)
Household size – N (%)		
2-3	33 (23.24)	39 (19.02)
4	45 (31.69)	72 (35.12)
5+	64 (45.07)	94 (45.85)
Ethnicity – N (%)		
Finnish	125 (88.03)	156 (76.10)
Foreign	17 (11.97)	49 (23.90)
Geographic region – N (%)		
Urban	115 (80.99)	177 (86.34)
Semi-urban	15 (10.56)	15 (7.32)
Rural	12 (8.45)	13 (6.34)

Notes: This table presents number of infections for healthcare workers' (under 18) children. **a** shows number of infections for children and adolescents aged from 3 to 18 years. **b** shows number of infections for children and adolescents aged from 3 to 12 years. **c** shows number of infections for adolescents aged from 3 to 18 years.

Supplementary Tables 5 and 6 document the number of infections for the estimation samples of healthcare workers, partners, and children. In the sample of healthcare workers, vaccinated healthcare workers had 22% of the infections and the rest of the infections (78%) were for unvaccinated healthcare workers (Supplementary Table 5, a). In the sample of partners, the partners of vaccinated healthcare workers had 34% of the infections (Supplementary Table 5, b). Similarly, for children, the children of vaccinated healthcare workers had 38% of the infections (Supplementary Table 6, a). Approximately 86% of the healthcare workers' infections were for women (Supplementary Table 5, a) and more than 87% of the partners' infections were for men (Supplementary Table 5, b). For children, the share of infections was almost balanced with respect to sex (Supplementary Table 6, a). In the samples of healthcare workers and partners, the largest share of infections was in two-person households (Supplementary Table 5, a-b) and in the sample of children, the largest share of infections was in households with more than five persons (Supplementary Table 6, a). In Supplementary Table 6, we group together household sizes 2 and 3, because of the Statistics Finland's data confidentiality requirements. More than 76% of the healthcare worker and partner infections were for Finnish individuals (Supplementary Table 5, a-b) and for children, over 73 % of infections were for Finnish individuals (Supplementary Table 6, a). Urban areas had more than 80% of infections in the samples of healthcare workers, partners, and children (Supplementary Table 5, a-b and Supplementary Table 6, a).

Supplementary Table 7 Healthcare worker infections by occupation code.

Occupation	Practical nurses (N=1 078)	Nurses and midwifes (N=309)	Physicians (N =48)	Dentists, audiologists and speech therapists (N=22)	Senior nursing officials and ward sisters (N=14)
Share of infections in sample (%)	73.28	21.01	3.26	1.50	0.95

Notes: This table presents the number and share of infections for healthcare worker occupations

Supplementary Table 7 further shows how infections are distributed between different occupation classes. Practical nurses (occupation code 532) had the largest share of infections and senior nursing officials and ward sisters (occupation code 222) had the smallest share. As expected, the number of infections arising from the occupation category of practical nurses and nurses and midwifes was the largest, because these occupations cover over 90% of the sample healthcare

workers (Supplementary Tables 4 and 7). Practical nurses were the only occupation category where the share of infections was substantially larger than the sample share of the occupation (73.28 % vs 66.38 %, Supplementary Tables 4 and 7).

Supplementary Table 8 Total number of individuals by follow-up week after the first dose.

**Follow-up week**	**0**	**1**	**2**	**3**	**4**	**5**	**6**	**7**	**8**	**9**	**10**	**11**	**12**
a													
Vaccinated (n)	112496	109635	98947	88820	85195	80449	78549	77297	76173	75097	73722	71636	67993
Unvaccinated (n)	152830	149103	140362	131449	122724	113854	105026	96054	87103	78212	69289	60389	51451
b													
Partners of vac. (n)	49219	48126	43965	40087	38778	37260	36754	36432	36111	35816	35361	34548	32893
Partners of unvac. (n)	79733	77826	73227	68529	63948	59343	54779	50092	45407	40729	36084	31503	26840
c													
Children of vac. (n)	37791	37043	34142	31164	30256	29244	28908	28690	28457	28277	27939	27270	25820
Children of unvac. (n)	67395	65731	61852	57906	53913	49987	46054	42131	38225	34275	30399	26527	22660
d													
Children of vac. (n)	26628	26106	23872	21707	21060	20303	20061	19889	19722	19563	19324	18832	17942
Children of unvac. (n)	37334	36378	34217	32068	29793	27622	25460	23283	21152	19032	16819	14619	12475

Notes: This table shows total number of individuals by follow-up week and vaccination status after the first dose. **a** shows the total number of healthcare workers. **b** shows total number of partners. **c** shows total number of children (3-12y). **d** shows total number of children (13-18y).

Supplementary Table 8 shows the total number of individuals (sample size) by follow-up week after the first dose. The sample sizes for the vaccinated and unvaccinated healthcare workers (Supplementary Table 8, a) were larger than the samples for their partners and children in each week (Supplementary Table 8, b-d). The weekly sample sizes are also larger for children aged 3-12 years old than for older children aged 13-18 years. Moreover, the sample sizes decrease over time after the first dose and are the lowest in the last week 12. The reason is that we can follow healthcare workers and their family members 12 weeks after the first dose only if their follow-up period started at the beginning of the observation period (vaccine administration in late December or in January).

B Supplementary Figures

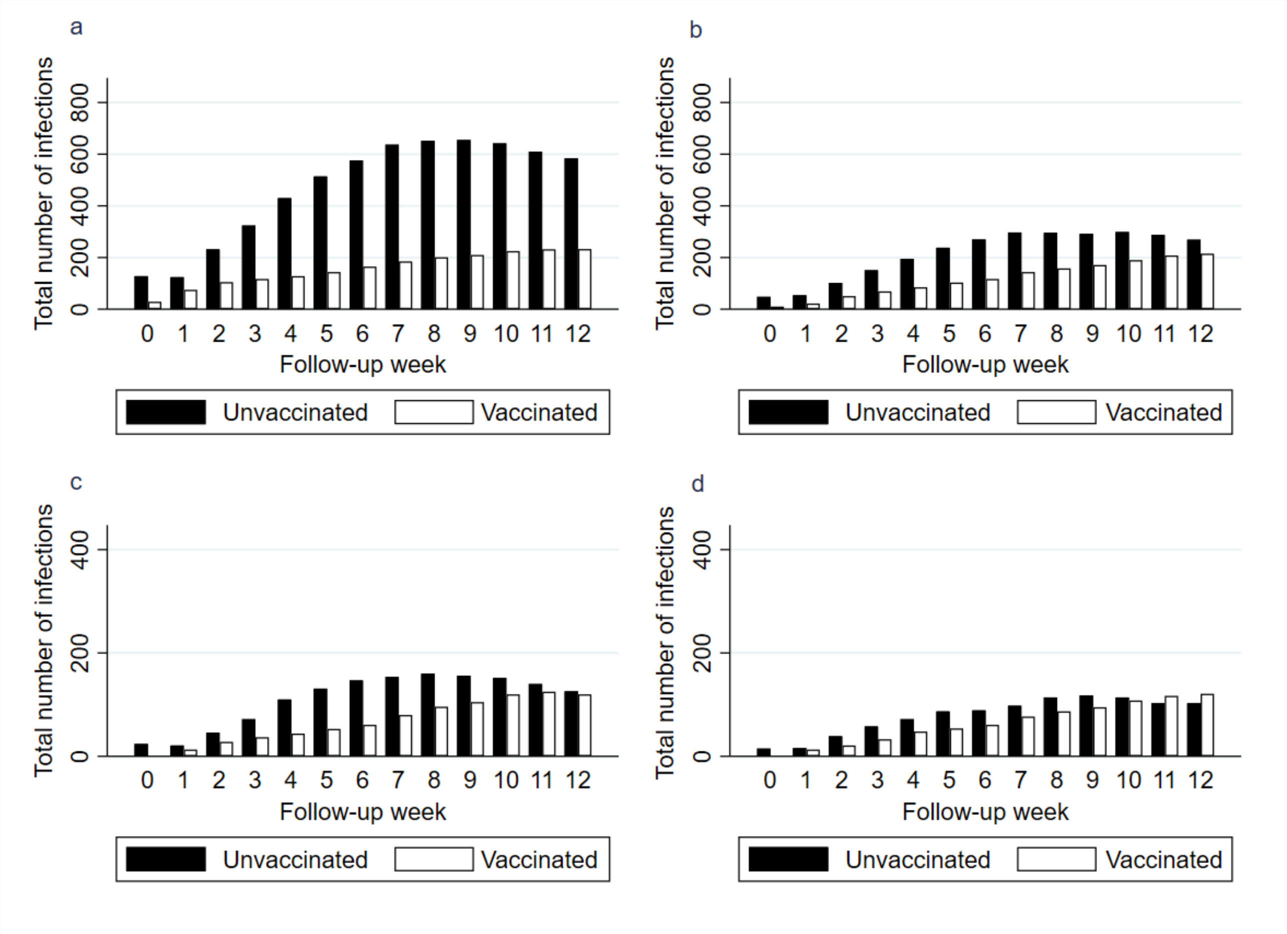

Supplementary Figure 1 Total cumulative number of infections. This figure plots the total cumulative number of infections by follow-up week after the first dose. **a** contains the healthcare workers. **b** contains their partners. **c** contains the household members aged 3-12. **d** the household members aged 13-18. The decrease in the total cumulative number of infections during the last weeks of the follow-up period reflects the corresponding decrease in the number of individuals (Supplementary Table 8).

Supplementary Figure 1 shows the total cumulative number of infections after the first dose for vaccinated and unvaccinated healthcare workers (Supplementary Fig. 1a), their partners (Supplementary Fig. 1b) and children (Supplementary Fig. 1c and 1d). As we focus on individuals with no prior infections before the follow-up period, the cumulative number of infections was very small during the first weeks, especially for children (in total, 34 for the children aged 3-12 years in week 1, for example). In later weeks, the cumulative number of infections was larger for vaccinated than unvaccinated individuals. As expected, the total cumulative number of infections increased over time after the first dose in the groups of healthcare workers, partners, and children. However, the total

infections decreased in the last weeks of the follow-up period, reflecting the corresponding decrease in the number of individuals (Supplementary Table 8).

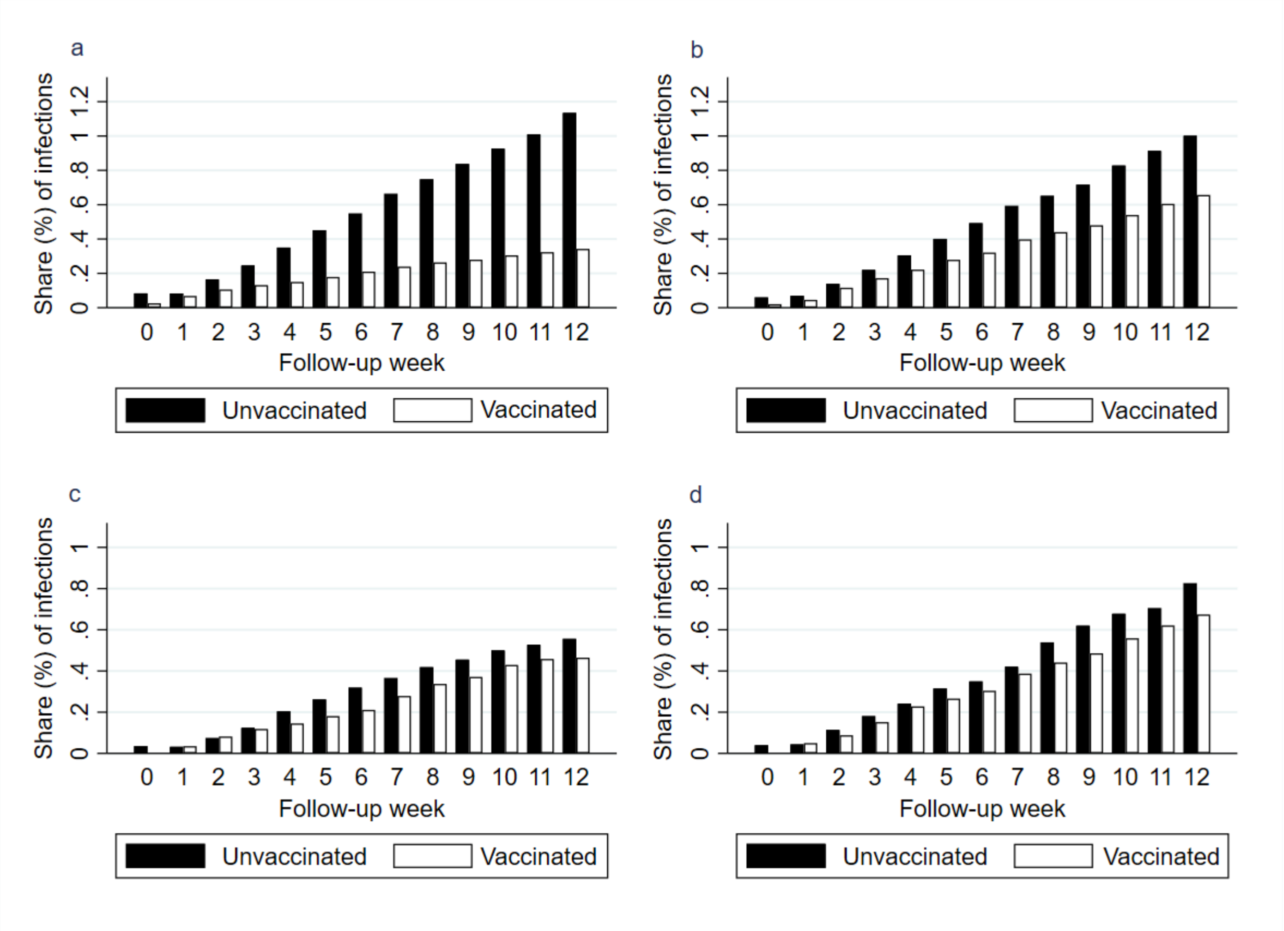

Supplementary Figure 2 Cumulative incidence of infections. This figure plots the cumulative incidence (population share) of infections by follow-up week after the first dose. **a** contains the healthcare workers. **b** contains their partners. **c** contains the household members aged 3-12. **d** the household members aged 13-18.

Supplementary Figure 2 instead shows that the cumulative incidence (population share) of infections increased gradually over time after the first dose. For example, the incidence of infections was 0.3% for vaccinated and 1.1% for unvaccinated healthcare workers in week 12, implying a 73% (approximately 100*(1-0.3/1.1)) unadjusted long-run estimate of direct vaccine effectiveness. In the same week, the incidence of the family members of vaccinated (unvaccinated) healthcare workers were 0.7% (1%) for the partners, 0.5% (0.6%) for the children aged 3-12 years, and 0.7% (0.8%) for the children aged 13-18 years. The results implied the unadjusted long-run estimate of indirect vaccine effectiveness of 30% for the partners, 16% for the children aged 3-12 years, and 13% for the children aged 13-18 years.

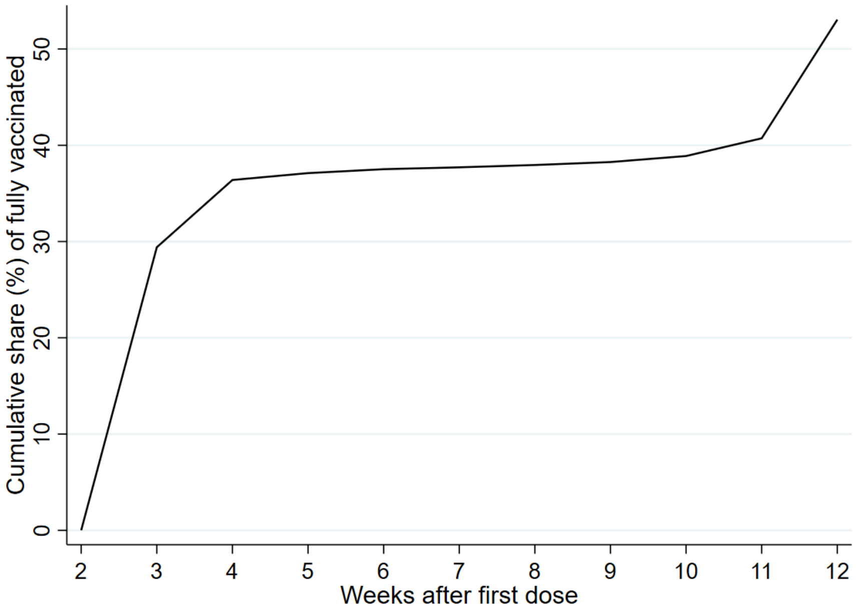

Supplementary Figure 3 The share of individuals with a second dose after the first dose. This figure plots the cumulative share of individuals with a second dose in our sample by week after obtaining the first dose. We calculated the shares by dividing the cumulative number of individuals with a second dose by the number of individuals with at least one dose.

The recommended interval between the first and second dose of the mRNA vaccines was initially three weeks but changed to 12 weeks in February 2021. Supplementary Figure 3 shows the average rate of receiving the second dose after the first dose in our sample. In the first two weeks after the first dose, the share of fully vaccinated individuals was zero, and in week three, it was approximately 30%. By week 12, over 50% of vaccinated healthcare workers had obtained their second dose.

C Supplementary Methods

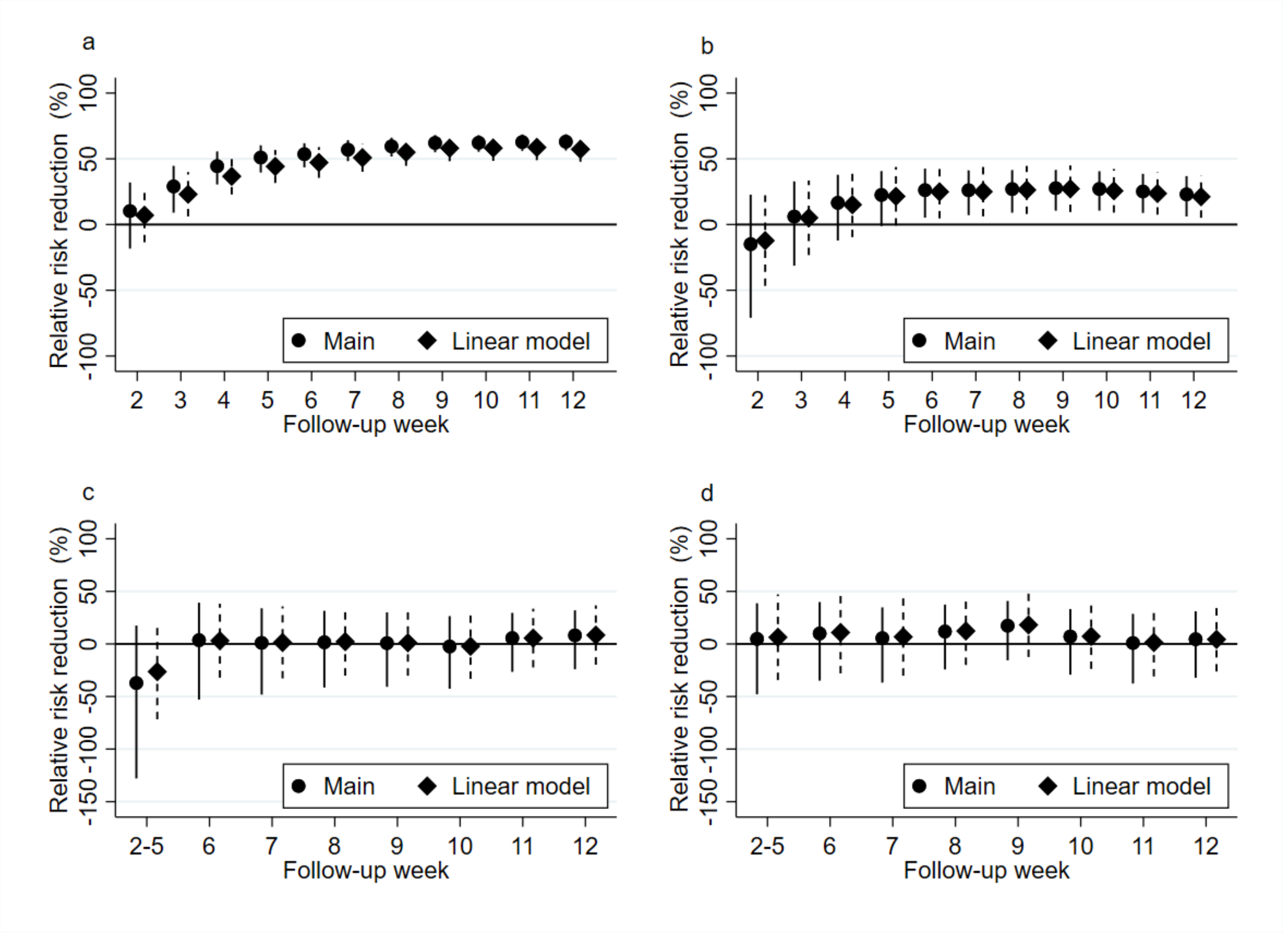

Supplementary Figure 4 Vaccine effectiveness in vaccinated individuals and unvaccinated family members using a linear probability model. This figure plots the vaccine effectiveness estimates by week after receiving the first dose of mRNA-based vaccination. The dependent variable is a polymerase chain reaction (PCR)-confirmed SARS-CoV-2 infection as recorded in the Finnish National Infectious Diseases Register. **a** shows the effectiveness estimates (relative risk reduction) for vaccinated individuals compared to the control group, which is constructed by randomly assigning the beginning of the follow-up period for unvaccinated individuals in the control group. N=265,326 healthcare worker observations. **b** shows the effectiveness estimates (relative risk reduction) for the unvaccinated partners (including cohabiting partners) of vaccinated individuals who lived in the same household as the vaccinated person as of December 31, 2019. N=128,952 partner observations. **c** shows the effectiveness estimates for the unvaccinated children aged 3-12 years of vaccinated individuals who lived in the same household as the vaccinated person as of December 31, 2019. N=105,186 child observations. **d** shows the corresponding effectiveness estimates for the children aged 13-18. N=63,962 adolescent observations. See section "Methods", "Estimation samples" for details of the sample restrictions. Coefficients in each panel are estimated using individual-date data collapsed to individual-week data. To construct the relative risk reduction estimates, we first regress the PCR-confirmed SARS-CoV-2 infection on the interaction of event week (follow-up week after vaccine administration) and vaccination status (vaccinated vs. unvaccinated). We then divide the regression coefficients by control group mean value. The relative risk reduction is presented as a percentage in data points ±95% confidence intervals. The solid black dots show relative risk reduction by week after receiving the first dose of mRNA-based vaccination. The error bars denote the 95% confidence intervals clustered at the individual-level using endpoint transformation of originally estimated confidence interval.

To study the sensitivity of our results for model specification, we estimated vaccine effectiveness using a linear probability model. The model is similar to that in equation (1) in the main text (Methods), but the outcome is simply the indicator of (cumulative) SARS-CoV-2 infection, y_{it}. To calculate the RRR in this case, we divided the β_l coefficient by the average probability of y_{it} in the control group in time-to-event week l. The 95% confidence intervals were calculated using the standard errors of the β_l coefficients, clustered at the individual-level. The results from the linear probability model for healthcare workers, their unvaccinated partners, and children are broadly consistent with the main findings (Supplementary Figure 4).
