# Supplementary material for: The indirect effect of mRNA-based COVID-19 vaccination on healthcare workers’ unvaccinated household members
Source: Nat Commun. 2022 Mar 4;13:1162. doi: 10.1038/s41467-022-28825-4 (PMC8897446; doi:10.1038/s41467-022-28825-4)
Supplement: Supplementary file 3 — Reporting Summary [file 41467_2022_28825_MOESM3_ESM.pdf]

nature portfolio

Corresponding author(s): Lauri Sääksvuori

Last updated by author(s): Jan 27, 2022

Reporting Summary

Nature Portfolio wishes to improve the reproducibility of the work that we publish. This form provides structure for consistency and transparency in reporting. For further information on Nature Portfolio policies, see our Editorial Policies and the Editorial Policy Checklist.

Statistics

For all statistical analyses, confirm that the following items are present in the figure legend, table legend, main text, or Methods section.

n/a	Confirmed	
☐	☒	The exact sample size (*n*) for each experimental group/condition, given as a discrete number and unit of measurement
☐	☒	A statement on whether measurements were taken from distinct samples or whether the same sample was measured repeatedly
☐	☒	The statistical test(s) used AND whether they are one- or two-sided<br>*Only common tests should be described solely by name; describe more complex techniques in the Methods section.*
☐	☒	A description of all covariates tested
☒	☐	A description of any assumptions or corrections, such as tests of normality and adjustment for multiple comparisons
☐	☒	A full description of the statistical parameters including central tendency (e.g. means) or other basic estimates (e.g. regression coefficient) AND variation (e.g. standard deviation) or associated estimates of uncertainty (e.g. confidence intervals)
☒	☐	For null hypothesis testing, the test statistic (e.g. *F*, *t*, *r*) with confidence intervals, effect sizes, degrees of freedom and *P* value noted<br>*Give P values as exact values whenever suitable.*
☒	☐	For Bayesian analysis, information on the choice of priors and Markov chain Monte Carlo settings
☒	☐	For hierarchical and complex designs, identification of the appropriate level for tests and full reporting of outcomes
☐	☒	Estimates of effect sizes (e.g. Cohen's *d*, Pearson's *r*), indicating how they were calculated

Our web collection on statistics for biologists contains articles on many of the points above.

Software and code

Policy information about availability of computer code

Data collection	No software was used to collect data by researchers. This study uses electronic health records stored in a SQL database.
Data analysis	Stata 16.1. All the code needed to reproduce the empirical results reported in this paper is available in an open science data repository (GitHub)

For manuscripts utilizing custom algorithms or software that are central to the research but not yet described in published literature, software must be made available to editors and reviewers. We strongly encourage code deposition in a community repository (e.g. GitHub). See the Nature Portfolio guidelines for submitting code & software for further information.

Data

Policy information about availability of data

All manuscripts must include a data availability statement. This statement should provide the following information, where applicable:

- Accession codes, unique identifiers, or web links for publicly available datasets
- A description of any restrictions on data availability
- For clinical datasets or third party data, please ensure that the statement adheres to our policy

The administrative healthcare and employment data used in this study are available from the Finnish Institute for Health and Welfare and Statistics Finland under restricted access due to Finnish data protection legislation. Healthcare data are also regulated under the Act on the Secondary Use of Health and Social Data (552/2019) and are, however, available upon reasonable request to and with the permission of Findata – Finnish Social and Health Data Permit Authority (https://findata.fi/en/). The Finnish Longitudinal Employer-Employee Data are available upon reasonable request to and with the permission of Statistics Finland (https://www.stat.fi). The authors are willing to assist in making data access requests.

Field-specific reporting

Please select the one below that is the best fit for your research. If you are not sure, read the appropriate sections before making your selection.

☒ Life sciences ☐ Behavioural & social sciences ☐ Ecological, evolutionary & environmental sciences

For a reference copy of the document with all sections, see nature.com/documents/nr-reporting-summary-flat.pdf

Life sciences study design

All studies must disclose on these points even when the disclosure is negative.

Sample size	Sample size was determined by the available electronic health care records (all administered mRNA-based vaccines and PCR-confirmed SARSCoV-2 infections from December 27, 2020 to April 25, 2021.
Data exclusions	Our analysis used three distinct estimation samples. The first sample was used in estimating the direct effect of the mRNA-based vaccinations (BNT162b2 by Pfizer-BioNTech or mRNA-1273 by Moderna). This sample consisted of working-age healthcare personnel (aged 15 - 74 years) excluding Pharmacists (22620), Environmental and occupational health and hygiene professionals (22630), Dieticians and nutritionists (22650), Health professionals not elsewhere classified (22690), Pharmaceutical assistants (53293), Equipment maintenance assistants (53292), Massage therapists and practical rehabilitation nurses (53294). The second sample was used in estimating the indirect vaccine effect on the unvaccinated partners of the healthcare workers. An individual was included in this sample if their partner is a healthcare worker and they had not been vaccinated during the sample period. The third sample was used in estimating the indirect vaccine effect on the children (aged 3 - 18) living in the same household as the healthcare worker. Children included in this sample are biological children of healthcare workers. An individual was included in this sample if they live in the same household as the healthcare worker and they had not been vaccinated during the sample period. Data exclusions are described in detail in Supplementary material.
Replication	This is a big-data observational study. All the code (Stata 16.1) needed to reproduce the empirical results reported in this paper is available in an open science data repository (GitHub). https://doi.org/10.5281/zenodo.5905898
Randomization	This is a big-data observational study. There was no randomization performed by the the researchers.
Blinding	This is a big-data observational study. There was no blinding performed by the the researchers.

Reporting for specific materials, systems and methods

We require information from authors about some types of materials, experimental systems and methods used in many studies. Here, indicate whether each material, system or method listed is relevant to your study. If you are not sure if a list item applies to your research, read the appropriate section before selecting a response.

Materials & experimental systems

n/a	Involved in the study	
☒	☐	Antibodies
☒	☐	Eukaryotic cell lines
☒	☐	Palaeontology and archaeology
☒	☐	Animals and other organisms
☐	☒	Human research participants
☒	☐	Clinical data
☒	☐	Dual use research of concern

Methods

n/a	Involved in the study	
☒	☐	ChIP-seq
☒	☐	Flow cytometry
☒	☐	MRI-based neuroimaging

Human research participants

Policy information about studies involving human research participants

Population characteristics	Our sample comprised a total of 265,326 healthcare workers, 128,952 unvaccinated partners of healthcare workers and 169,148 unvaccinated children of healthcare workers. The mean ages of the healthcare workers, their spouses, and children were 44 (SD 14), 45 (SD 12) and 11 (SD 5) years, respectively. Most healthcare workers (86.6%) in our sample were women. A total of 112,496 healthcare workers (42.4%) obtained at least one dose of mRNA-based vaccine during the period from December 27, 2020 to April 25, 2021. The number of doubly vaccinated healthcare workers during the same time period was 63,986 (24.0%). The total number of PCR-confirmed SARS-CoV-2 infections in our sample was 1471 (0.55%), 782 (0.61%), and 820 (0.48%) for healthcare workers, partners and children, respectively.
Recruitment	This is an observational study involving electronic health care records analysis.
Ethics oversight	This research complies with all the relevant ethical regulations. The final data used in the study was de-identified and

Ethics oversight	therefore research does not constitute human subject research. Ethical approval was waived by the Institutional Review Board of the Finnish Institute for Health and Welfare (IRB: 00007085).

Note that full information on the approval of the study protocol must also be provided in the manuscript.
